# Metaphorical Language in Paediatric Vaccine Communication: Framing Risk and Building Trust Among Children and Adolescents

**DOI:** 10.1111/apa.70534

**Published:** 2026-04-08

**Authors:** Fabiana Nuccetelli, Valeria Gabellone, Pier Luigi Lopalco

**Affiliations:** ^1^ Department of Experimental Medicine University of Salento Lecce Italy

**Keywords:** health communication, metaphorical framing, paediatric vaccine communication, parental decision‐making, risk perception, vaccine confidence

## Abstract

**Aim:**

To examine the effectiveness of metaphorical language in framing risk and building trust among parents, children and adolescents in paediatric vaccine communication.

**Methods:**

A narrative synthesis was conducted using empirical studies, institutional reports from the Istituto Superiore di Sanità, European Centre for Disease Prevention and Control, and National Institute for Health Research, alongside an original survey involving 2681 Italian adolescents, providing preliminary results. The survey compared three message framings: war metaphors, protective/caring metaphors and neutral factual statements.

**Results:**

Protective metaphors emphasising safety, care and collective responsibility achieved the highest Vaccine Confidence Index scores (mean 9.1), significantly outperforming both war metaphors and neutral messages (*p* < 0.01). While war metaphors may initially mobilise urgency, they risk inducing anxiety, resistance and reduced perceived personal agency, particularly among younger audiences.

**Conclusion:**

Integrating age‐appropriate protective and caring metaphors into paediatric vaccine communication enhances trust and vaccine acceptance. Future campaigns should prioritise language that fosters safety, community and personal responsibility while avoiding metaphors likely to trigger fear or alienation.

## Key Points

- This review was conducted to understand how metaphors influence vaccine confidence among children, adolescents and parents.
- Protective and caring metaphors improved trust and acceptance more effectively than war metaphors or neutral messages.
- Incorporating protective metaphors into paediatric vaccine campaigns can enhance trust, reduce anxiety and support informed decision‐making in clinical and public health practice.

## 1 Introduction

Effective risk and vaccine communication in paediatric care is essential to protect children and adolescents from infectious diseases and promote public health. During recent health crises, such as the COVID‐19 pandemic, the use of metaphorical language in public health messaging has increased substantially, serving as a powerful tool to shape understanding, attitudes and behaviours [1, 2]. Metaphors can simplify complex scientific information and evoke emotional responses, making messages more relatable and memorable [3]. However, their impact varies depending on the metaphor chosen, the age of the audience and the cultural context [4, 5]. This review explores the role of metaphorical language in paediatric vaccine communication, focusing on its capacity to frame risk and build trust among parents, children and adolescents. It combines evidence from the literature with data from an original adolescent survey to inform age‐appropriate communication strategies that enhance vaccine confidence [6, 7].

## 2 Methods

This narrative review combined empirical studies, institutional reports from the Istituto Superiore di Sanità, European Centre for Disease Prevention and Control, and National Institute for Health Research, with data from an original survey of 2681 Italian adolescents. Inclusion criteria encompassed empirical studies, institutional reports and original survey data directly addressing metaphor use in paediatric vaccine communication. Exclusion criteria included studies not involving paediatric populations, non‐health‐related metaphors and articles without primary data or relevant analysis.

A systematic search strategy was applied across PubMed, Scopus, Web of Science and PsycINFO. The search focused on literature published between 2010 and 2024 to capture contemporary shifts in vaccine communication. Keywords used included ‘paediatric vaccine communication’, ‘metaphor’, ‘risk perception’, ‘trust’, ‘health framing’, ‘parental decision‐making’ and ‘adolescent decision‐making’. After removing duplicates, 142 records were screened by title and abstract; 35 full‐text articles and 4 institutional reports were eventually included based on their direct relevance to metaphorical framing in paediatric settings. Boolean operators (AND/OR) were used to refine results. Additionally, reference lists of included studies and grey literature (conference abstracts, policy reports) were screened to ensure comprehensiveness.

## 3 Results

### 3.1 The Role of Metaphors in Paediatric Health Communication

Metaphors function as cognitive and emotional tools, enabling audiences to understand abstract or complex concepts through familiar imagery [1, 3]. In paediatric health, metaphors can help children and parents grasp the importance of vaccination and preventive behaviours [2, 5]. From a psychological perspective, metaphors act as frames that guide interpretation and influence reasoning [4]. They activate associative networks, allowing children to map unfamiliar biomedical concepts onto everyday experiences, thereby reducing uncertainty and providing a sense of control [8].

Developmental psychology highlights that children between 8 and 14 years are particularly receptive to protective and care‐oriented metaphors, as these align with their emotional and social identity formation [9]. Thus, metaphors not only facilitate comprehension but also foster trust and positive emotional engagement, key determinants of vaccine confidence [10].

### 3.2 War Metaphors: Strengths and Limitations

War metaphors, such as ‘fighting the virus’ or ‘battling the pandemic,’ have been widely used to mobilise urgency and collective action during health crises [11, 12]. They can initially capture attention and may foster solidarity in contexts of acute emergency, creating a sense of shared mission and resilience [13]. However, they also risk inducing anxiety, fear and helplessness, especially among children and adolescents who may feel excluded from the ‘battle’ [14]. Young people often disengage from combative language, perceiving it as alienating or paternalistic [6]. Moreover, such framing can imply a loss of agency, focusing on conflict rather than cooperation [13].

### 3.3 Protective and Caring Metaphors: A More Effective Framing

Protective and caring metaphors emphasise safety, nurturing, community and personal responsibility, creating a more positive communication environment [15]. These metaphors resonate strongly with parents and children, reducing anxiety and enhancing trust in healthcare [16]. Campaigns portraying vaccines as ‘shields’ or ‘acts of guardianship’ have shown increased engagement and vaccine acceptance [7, 17]. Nevertheless, they are not universally effective. Adolescents striving for autonomy may perceive ‘care’ metaphors as paternalistic, while individuals with strong independence‐oriented values might resist messages that frame vaccination primarily as protection by authority figures [10, 15]. This underlines the need for tailoring metaphors not only to developmental stage but also to cultural and psychosocial identity. To clarify the application across target groups: for younger children (8–11 years), ‘protective’ metaphors (e.g., a shield) are most effective as they align with concrete operational thinking [9]. For adolescents (12–18 years), metaphors emphasising ‘collective responsibility’ and ‘social agency’ are preferable to avoid perceptions of paternalism [10]. For parents, metaphorical framing serves as a bridge to simplify risk–benefit trade‐offs, supporting informed ‘parental decision‐making’ by focusing on long‐term guardianship rather than immediate fear [18].

### 3.4 Case Study: Metaphorical Framing and Vaccine Confidence in Italian Adolescents

A recent study involving 2681 Italian adolescents compared war metaphors, protective/caring metaphors and neutral factual messages [6]. Using a randomised controlled design, researchers measured the Vaccine Confidence Index (VCI) before and after exposure.

The VCI is a composite measure assessing trust in vaccine safety, efficacy and importance on a 0–10 scale. It integrates both cognitive and emotional dimensions of trust, making it a sensitive proxy for attitudes towards vaccination [19].

Results showed that protective metaphors yielded the highest VCI scores (mean 9.1), significantly exceeding those from war metaphors and neutral messages (*p* < 0.01). This suggests that age‐appropriate protective metaphors enhance both trust and perceived agency in vaccination decisions.

Psychologically, these metaphors are effective because they tap into emotional drivers such as care, belonging and responsibility, particularly salient in adolescence [10, 16]. However, it remains unclear whether these effects translate into long‐term behavioural change. Further longitudinal research is needed to examine whether positive shifts in vaccine confidence are sustained over time and result in increased vaccine uptake.

## 4 Conclusions

### 4.1 Clinical and Communication Implications

Paediatricians, public health professionals and educators should tailor communication strategies to developmental stages [18]. Age‐appropriate metaphors can foster trust without provoking anxiety, empowering both parents and adolescents. Messages should emphasise protection, community support and shared responsibility while avoiding culturally inappropriate or overly paternalistic framing [14, 20].

### 4.2 Conclusion and Future Directions

Protective and caring metaphors consistently outperform war metaphors in fostering vaccine confidence among young populations [6, 7]. Age‐appropriate metaphorical framing enhances trust, reduces hesitancy and supports informed decision‐making.

This review has some limitations, including its narrative design, which may be subject to selection bias, and the preliminary nature of the survey data, which requires further validation. Additionally, findings from Italian adolescents may not be directly generalisable to other cultural contexts.

Furthermore, it is crucial to acknowledge that vaccine acceptance is highly context‐specific. The effectiveness of these linguistic tools may vary depending on whether the vaccine is mandatory or voluntary, part of a routine schedule or an emergency pandemic response. For instance, during an acute outbreak, the urgency of ‘war’ metaphors might find temporary utility, whereas routine paediatric care benefits more from sustained ‘trust‐building’ protective frames [11, 12].

Future vaccine campaigns should strategically integrate protective metaphors, while avoiding language that induces fear or alienation. Interdisciplinary research is warranted to explore metaphor use across cultures and age groups [20].

## Author Contributions

**Fabiana Nuccetelli:** writing – original draft, methodology, formal analysis. **Pier Luigi Lopalco:** conceptualization, validation, writing – review and editing, supervision. **Valeria Gabellone:** writing – original draft, methodology, formal analysis.

## Conflicts of Interest

The authors declare no conflicts of interest.

## Data Availability

Data sharing not applicable to this article as no datasets were generated or analysed during the current study.
